# An Enhanced Temperature Control Approach to Simulate Profile Extrusion

**DOI:** 10.3390/polym16070904

**Published:** 2024-03-25

**Authors:** João Vidal, João Miguel Nóbrega

**Affiliations:** 1 Soprefa-Componentes Industriais SA, R. Alfredo Henriques, 4520-909 Mosteiró, Portugal; 2 Institute for Polymers and Composites, University of Minho, Campus de Azurém, 4800-058 Guimarães, Portugal; mnobrega@dep.uminho.pt

**Keywords:** profile extrusion dies, temperature control, boundary condition, OpenFOAM

## Abstract

Thermoplastic extrusion, a widely used method for processing thermoplastic materials, requires precise temperature control to ensure product quality. However, existing computer-aided engineering tools often oversimplify the temperature distribution calculations, leading to additional discrepancies between simulations and the actual processes. This study introduces a novel multi-region modeling approach to address this issue. By employing realistic temperature control conditions, the methodology overcomes the limitations of current numerical modeling tools. The key contributions include the development of a transient, incompressible, non-isothermal solver integrated into the OpenFOAM computational library and the implementation of a specialized boundary condition that emulates Proportional-Integral-Derivative (PID) control using real-time thermocouple measurements. The findings highlight temperature deviations at the flow channel walls and total pressure drop while demonstrating a smaller impact on velocity and flow uniformity at the outlet under steady-state conditions. This research substantially advances the understanding of thermal dynamics in extrusion processes, offering crucial insights for enhancing temperature control and laying the groundwork for more effective and precise operational strategies.

## 1. Introduction

The polymer extrusion process is a critical industrial manufacturing technique used across various economic sectors to produce profiles for construction, automotive, and other applications [1]. The process involves five main components: the extruder, die, calibration and cooling, haul-off, and cutting, each with a defined function in the extrusion process [2]. Temperature plays a crucial role in achieving high-quality output products [3], particularly in the die, where effective heating is essential. Cartridge heaters and band heaters are commonly used to achieve temperature control in the die [4,5]. To achieve stable temperature fields in the die, various control algorithms have been developed, including Proportional-Integral-Derivative (PID) and fuzzy logic algorithms [6,7].

Computational simulation is an invaluable tool in understanding and optimizing the extrusion process. Open-source software, such as OpenFOAM^®^, has enabled companies and researchers to perform cost-effective numerical modeling, implementing complex physical models with ease [8,9].

For modeling single-screw extruders, researchers have used the Finite Element Method (FEM) and imposed temperatures at the barrel and screw walls [10,11]. Similarly, for twin-screw extruders, models incorporating heat transfer coefficients and temperature at the barrel and screw materials have been developed [12,13].

The extrusion die, being the crucial component in shaping the molten material, has obviously garnered attention for simulation. Researchers have developed methodologies imposing boundary conditions for temperature at the outer surface of the flow channel and considering the torpedo as insulated [14,15]. More recent work explored complex three-dimensional shapes but did not consider temperature effects on the flow [16]. Extrudate swell, a significant phenomenon in polymer extrusion, has also been studied using methods like the Boundary Element Method (BEM) [17].

Design and optimization of polymer profile extrusion dies traditionally relied on trial-and-error based on designers’ expertise, leading to increased costs due to numerous iterations [1,18]. Numerical tools have been proposed to automate die design optimization, combining Finite Element Analysis (FEM) and Flow Analysis Network [19]. Studies using the Finite Volume Method (FVM) aimed to optimize slit extrusion dies, considering temperature effects by imposing temperatures at the inlet and flow channel walls [20,21]. Commercial software like PolyXtrue and Polyflow has been used to simulate polymer extrusion die flow channels [22,23], but the calculations are limited to the flow channel, with certain assumptions at its surface. The open-source OpenFOAM^®^ computational library has also been employed in modeling the polymer extrusion process in a similar manner and considering temperature on torpedo walls, or utilizing insulation [24,25]. This assumption has been defined based on intuition, but its validity was never verified before.

In the context of modeling profile extrusion, all available tools typically assume that either the temperature or the heat flux is imposed at the flow-channel surface. However, in practical applications, temperature control in extrusion is often achieved using thermocouples that measure the temperature in the metallic tool at a finite distance from the flow channel surface. Indeed, Abeykoon [6] introduced a novel method for measuring die melt temperature profiles. Yet, this method affects the melt flow and is only applicable in very specific open locations, which excludes the extrusion die flow with varying flow channels. As a result, it cannot fully replace the currently available temperature control systems.

Given these considerations, to improve the usefulness of computational simulation tools in understanding and optimizing the profile extrusion process, it is critical to recognize and address the limitations that arise from oversimplified temperature modeling. Such simplifications in temperature modeling may lead to inaccuracies in predictions and limit the effectiveness of efforts to enhance existing temperature control strategies. In this work, the authors present a new simulation code, which can model an incompressible, transient, multi-region solver and a realistic temperature control boundary condition implemented in the OpenFOAM computational library that replicates the real systems behavior.

The article is organized as follows: in Section 2, the governing equations, the solver, and the boundary condition implementation are presented. In Section 3, the industrial case study and different possible approaches for modeling are presented. In Section 4, the result presentation, analysis, and discussion are performed. Finally, in Section 5, the main conclusions of the work are presented.

## 2. Computational Framework

### 2.1. Solver Implementation

The multi-region solver implementation draws its foundation from pimpleFoam [26], a solver that employs the PIMPLE method for coupling pressure and velocity while seamlessly integrating PISO and SIMPLE methodologies [27].

The equations solved are the momentum conservation (1),

(1) $$\frac{𝜕u}{𝜕t}+\nabla \cdot (\rho uu)=-\nabla p+\nabla \cdot \tau$$

The mass conservation (2),

(2) ∇·u=0,

To consider the influence of temperature, it is imperative to incorporate the energy conservation equations, both in the fluid (3) and within the solid (4),

(3) $$\frac{𝜕T}{𝜕t}+\nabla \cdot \left(uT\right)-\nabla \cdot \left(\alpha \nabla T\right)=\frac{1}{c_{p}}\tau :\nabla u,$$

(4) $$\frac{𝜕T}{𝜕t}-\nabla \cdot \left(\alpha \nabla T\right)=0,$$

In the given equations, the variables hold the following meanings: T represents the temperature, ρ the fluid density, **u** the velocity vector, p the pressure, τ the deviatoric stress tensor, $c_{p}$ the specific heat, and α symbolizes thermal diffusivity. Within Equation (3), the last term on the right-hand side (τ:∇u) represents the viscous dissipation contribution.

**Figure 1:**
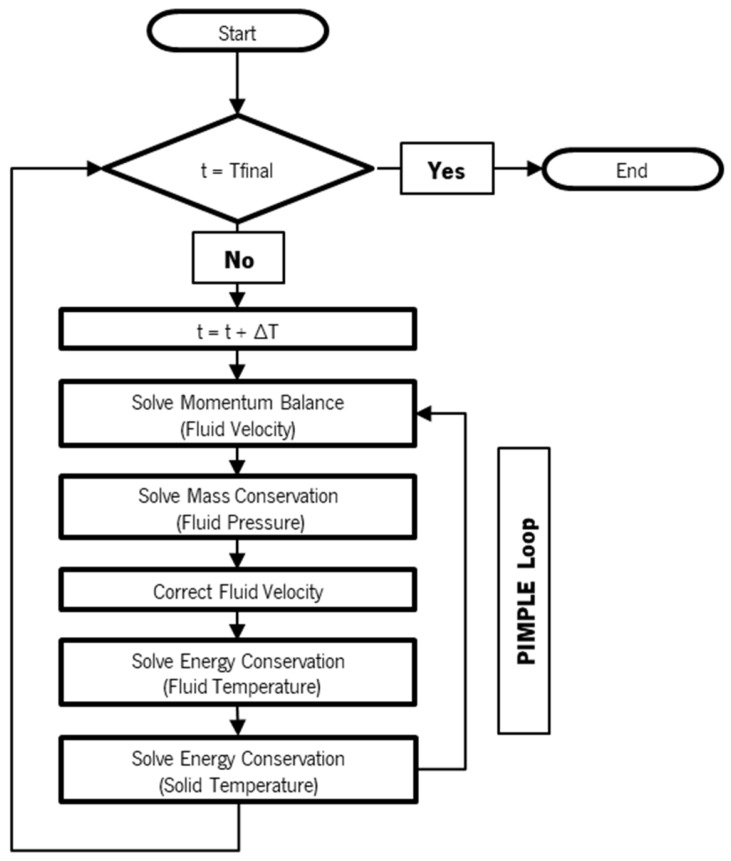
Solver flowchart.

As illustrated in the flowchart presented in Figure 1, for each time step, the developed transient solver solves the momentum balance equations, mass conservation, and energy conservation equations for the fluid and, subsequently, the energy conservation equation for the solid. The process is iteratively repeated until the convergence, measured by the residuals of each equation, is achieved.

### 2.2. Boundary Condition Implementation

The implementation of the heater control boundary condition performed in this work was based on the externalWallHeatFluxTemperature [28] boundary condition, which imposes a heat flux at the external wall.

The implementation of the PID algorithm [29] is based on the following equation:

(5) $$PID\left(t\right)=K_{p}e\left(t\right)+K_{i}\int e\left(t\right)dt+K_{d}\frac{de}{dt}$$

where, $K_{p}$, $K_{i}$ and $K_{d}$ are, respectively, the proportional, integral, and differential gains. Moreover, $e\left(t\right)$, the difference between the probe and objective values is given by the following:

(6) $$e(t)=T_{probe}-T_{obj}$$

Subsequently, following the incorporation of the PID equation, a conditional response was introduced to the boundary condition. Specifically, when the PID(t) value is below 0, a fixed Gradient boundary condition is employed to impose the input power from the heater. Conversely, if the PID(t) function value equals or surpasses 0, a Robin boundary condition is employed, which represents the heat flux through natural convection.

## 3. Case Studies

**Figure 2:**
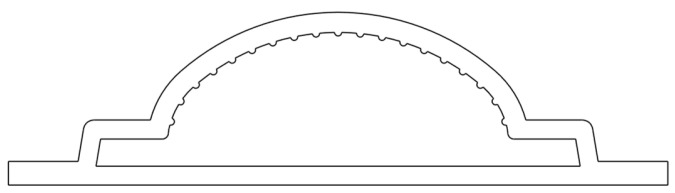
Industrial case study profile cross-section.

**Figure 3:**
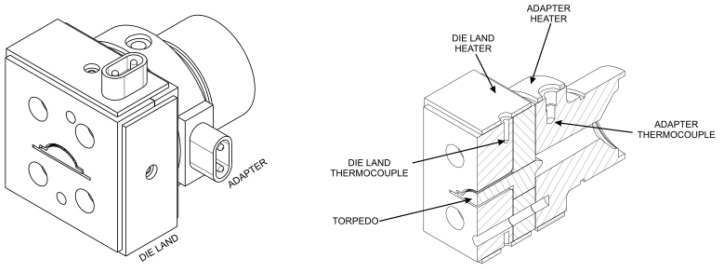
Extrusion die regions (**left**) and die heating control elements (**right**).

The assessment of the developed code was performed with an industrial case study, which focuses on the production of an LED encasing profile, whose cross-section, which is approximately 40 mm wide, is shown in Figure 2. The extrusion die employed in this investigation comprises two heaters to control the temperature in two different regions of the die, the Adapter and the Die Land, as illustrated in Figure 3 (left). Furthermore, as shown in Figure 3 (right), each heater is equipped with an independent thermocouple that controls the temperature in the corresponding region.

The LED encasing profile in the industrial case study is made of polycarbonate material (Trirex 3027 U[M1], supplied by Samyang Corporation, Seoul, Republic of Korea). For the constitutive model, the Bird–Carreau model coupled with the Arrhenius Law, given by Equations (7) and (8), was employed. The former considers the shear rate, $\dot{\gamma }$, dependence while the latter the temperature, T, dependence.

(7) $$\eta \left(\dot{\gamma },T\right)=a_{T}\eta_{\infty }+\frac{a_{T}\left(\eta_{0}-\eta_{\infty }\right)}{{\left(1+{\left(a_{T}\lambda \dot{\gamma }\right)}^{2}\right)}^{\frac{1-n}{2}}}$$

(8) $$a_{T}=\exp\left(\frac{E}{R}\left(\frac{1}{T}-\frac{1}{T_{0}}\right)\right)$$

For the simulation, the physical parameters provided in Table 1 were obtained from Aali et al. [30], where the same material was characterized with capillary and parallel plate rheometries. The die material properties are provided in Table 2.

**Figure 4:**
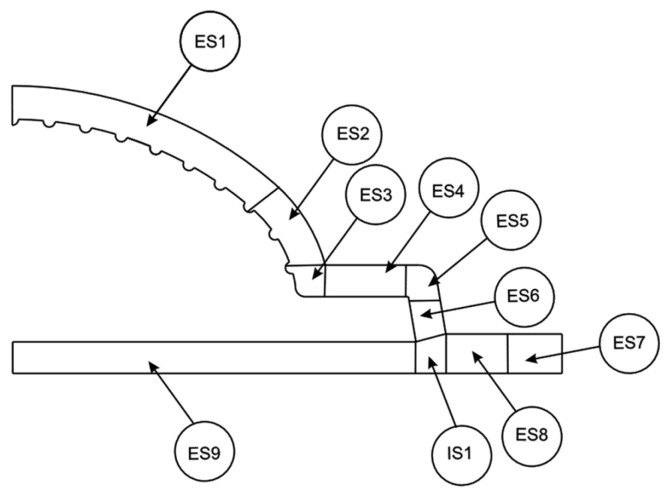
Flow channel outlet cross-section division in elemental (ES) and Intersection (IS) sections.

To assess the flow distribution at the flow channel outlet, and following the methodology proposed by Rajkumar et al. [24], the cross-section was divided into elemental (ES) and Intersection (IS) sections, as illustrated in Figure 4. The extrusion die performance was quantified by an overall objective function, given Equation (9), that combines the contribution of the individual sections’ objective function (Equation (10)), each of which is weighted based on the corresponding outlet section area ($A_{\mathrm{target},i}$), being $A_{\mathrm{target},tot}$ the total cross-section area. In Equation (10), $Q_{i}$ and $Q_{\mathrm{target}}$, represents, respectively, the actual and target flow rates in each section [24].

(9) $$F_{obj}=\frac{\sum_{ES+IS}\left\|F_{obj,i}\right\|A_{\mathrm{target},i}}{A_{\mathrm{target},tot}}$$

(10) $$F_{obj,i}=\frac{\frac{Q_{i}}{Q_{\mathrm{target}}}-1}{\max\left(\frac{Q_{i}}{Q_{\mathrm{target}}},1\right)}$$

To assess the implemented code in detail, three different modeling approaches were employed, which are described in the following subsections.

### 3.1. Conventional Approach

**Figure 5:**
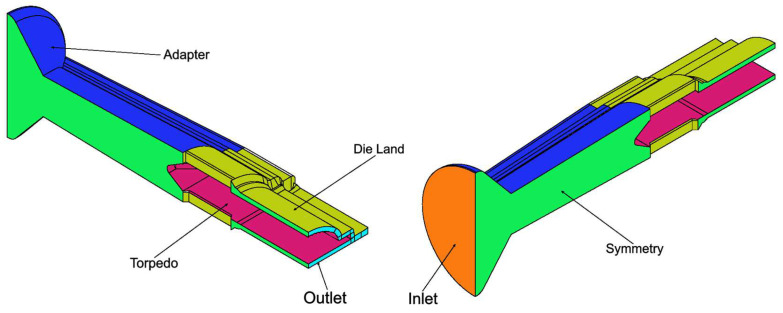
Conventional approach flow channel geometry with the indication of the considered boundary patches.

This case study mimics the conventional simulation approach, described in Section 1, usually employed to model these cases. To simulate the process with the approach, just the flow channel is considered, and its external surface is divided into different regions (named as patches), as shown in Figure 5, on which boundary conditions are applied in accordance with Table 3 for all the fields being calculated: velocity, pressure, and temperature. In this case, following the conventional modeling approach described in Section 1, the Temperatures imposed at the patches Inlet, Adapter, and Die Land were the ones set up in the real system. Moreover, the Inlet velocity was defined to achieve an average velocity of 3 m/min at the Outlet, which was the actual production velocity.

### 3.2. Multi-Region Approach

**Figure 6:**
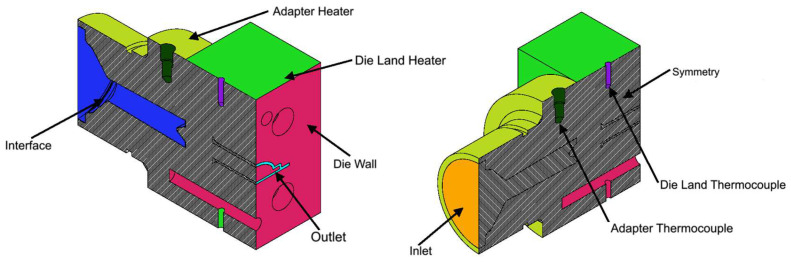
Multi-region approach geometry with the indication of the considered boundary patches.

The novel approach implemented to simulate the flow considers both the metallic tool and the flow channel and a larger number of patches at the geometry surface together with the interface, as shown in Figure 6. The corresponding boundary conditions are presented in Table 4. In this case, both at the Adapter Heater and Die Land Heater patches, the novel boundary condition, described in Section 2.2, was imposed. Accordingly, the operation of both heaters is controlled by the average temperature calculated at the respective thermocouple patches: Adapter Thermocouple and Die Land Thermocouple.

### 3.3. Mixed Approach

The third and last case study considered aims at following the conventional approach (Section 3.1), but with adjusted temperature on patches Adapter and Die Land. For that, based on the calculations performed with the multi-region approach (Section 3.2), the average temperature at those patches was computed after reaching quasi-steady state conditions, being the obtained values imposed at the respective patches, as presented in Table 5. The geometry used in the case study is the same as the one used for the conventional approach, which is illustrated in Figure 5.

### 3.4. Computational Mesh

**Figure 7:**
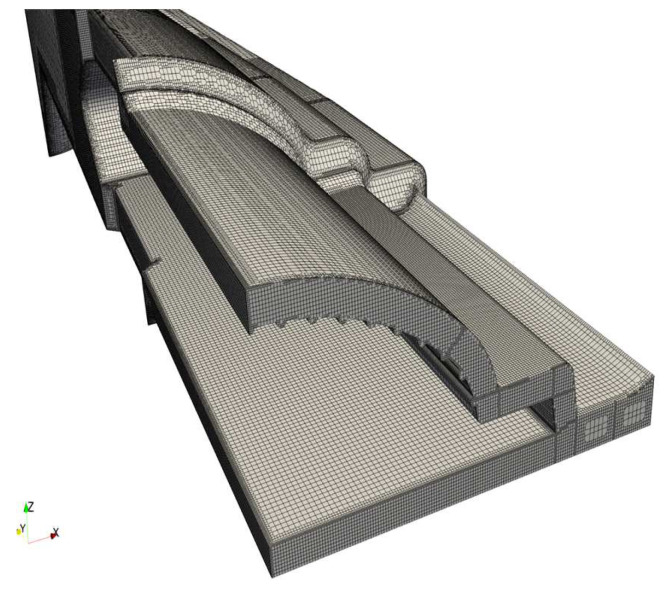
Conventional and Mixed approaches mesh.

**Figure 8:**
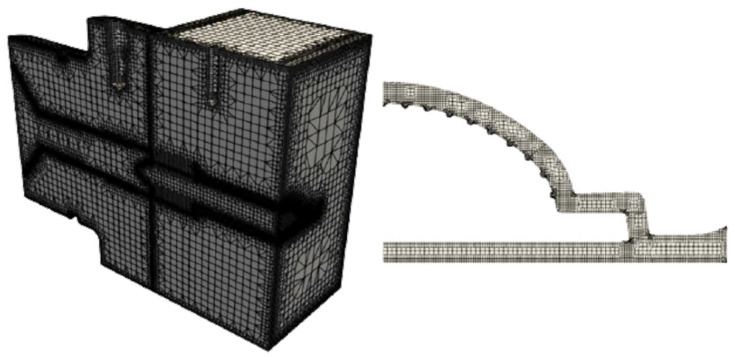
Multi-region approach mesh.

Prior to initiating the numerical studies, a mesh sensitivity study was conducted to determine the necessary refinement level. The mesh generation process was carried out using the OpenFOAM utility snappyHexMesh for both the conventional and multi-region approaches. The meshes selected for carrying out the studies are presented in Figure 7 and Figure 8.

## 4. Results and Discussion

**Figure 9:**
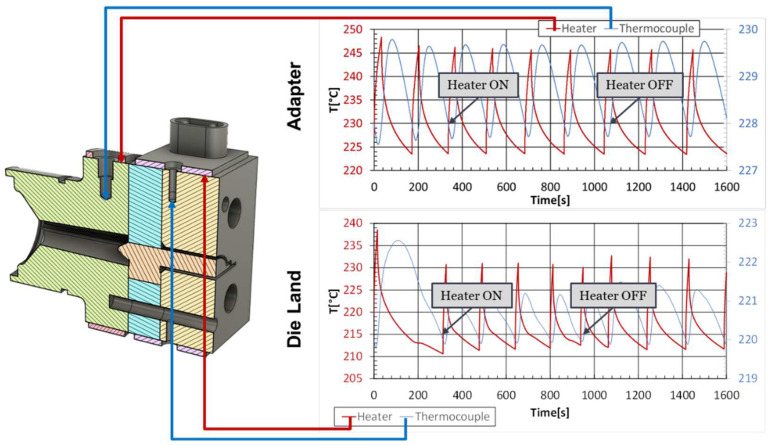
Evolution of the average temperature at the heaters and respective thermocouples for the multi-region approach case study.

Figure 9 illustrates the average temperature evolution of the heaters and their respective thermocouples in both the Adapter and Die Land regions for the multi-region approach case study. These results clearly demonstrate the influence of the heater’s state on the temperature of both the heaters and their respective thermocouples. When the heater is turned on, the temperature in that area increases immediately, while the opposite occurs when it is disconnected, while at a lower cooling rate, since the heat removal occurs by natural convection. Due to the finite distance between the thermocouple and the heater, there is a time delay between the actions of the heater and the corresponding effect measured by the thermocouple.

**Figure 10:**
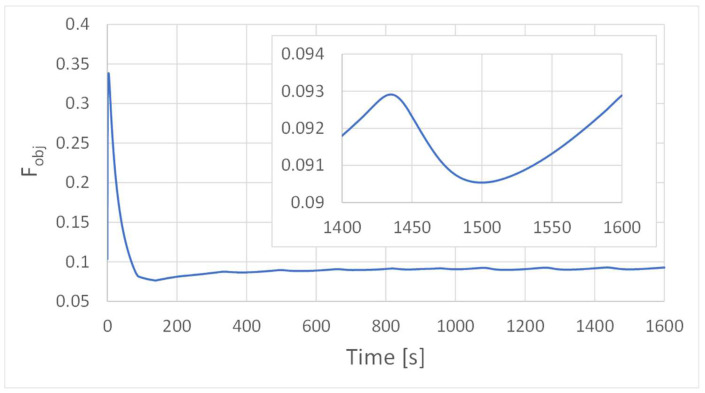
Evolution of F_obj_, Equation (10), along with the simulation for the multi-region approach case study.

The evolution of the objective function, Equation (10), which measures the flow distribution uniformity, is illustrated in Figure 10 for the multi-region approach case study. As shown after a period of 300 s, it becomes clear that the objective function stabilizes, reaching almost steady-state conditions, and the influence of the unsteady heater operation diminishes.

**Figure 11:**
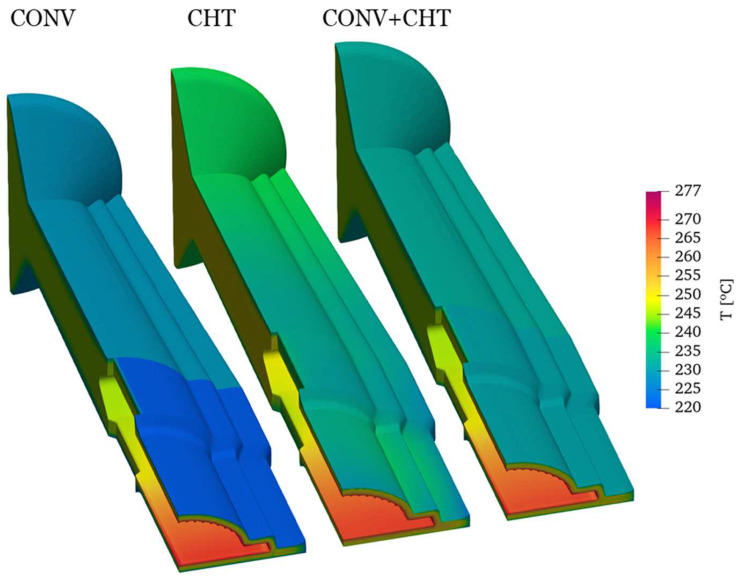
Comparison of temperature field for the three case studies considered.

The results from Figure 11 show the temperature distribution across three different case studies: the conventional (CONV), multi-region (CHT), and mixed (CONV+CHT) approaches. It is observed that the multi-region (CHT) approach produces a higher and more uniform temperature distribution along the outer wall of the flow channel compared to the conventional (CONV) approach. Additionally, the temperature distribution on the outer surface of the mandrel is slightly higher in the CHT approach, especially near the inlet. This difference is attributed to the higher temperature of the adapter walls, which leads to an elevated polymer temperature.

Interestingly, despite these differences, the results indicate that heat transfer within the mandrel is negligible. This is likely due to minimal heat loss through the front surfaces of the mandrel and low heat transfer through the spider legs, the connecting elements between the mandrel and the die. As a result, assuming an insulated patch in the conventional (CONV) and mixed (CONV+CHT) approaches seems to be a reasonable approximation. While this assumption may not exactly match the predictions of the CHT approach, it provides a close approximation.

**Figure 12:**
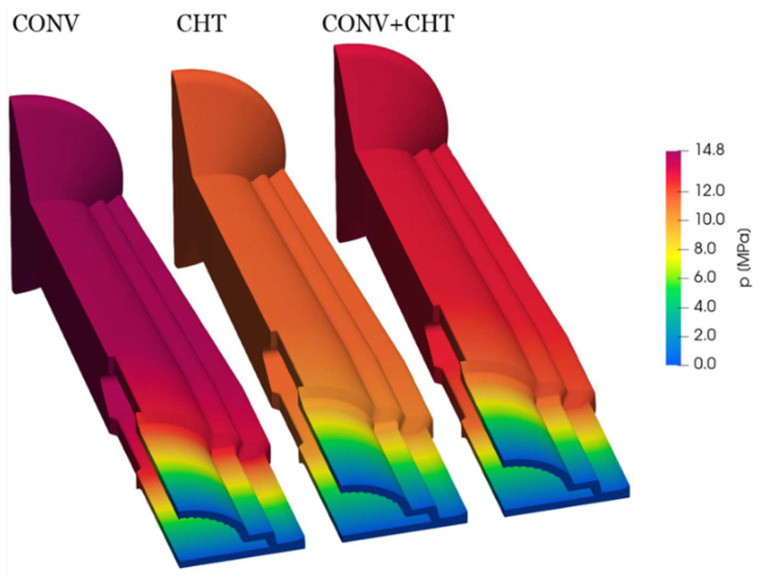
Comparison of pressure field for the three case studies considered.

In Figure 12, two important observations can be made about the pressure field analysis. Firstly, it is clear that the higher temperatures predicted in the CHT approach are associated with a lower pressure drop. This effect is especially noticeable in the Adapter region of the flow channel. The Adapter cross section is thicker, and therefore, the fluid has a lower velocity magnitude, resulting in a very small pressure gradient along this region due to continuity. As the flow channel becomes narrower, the velocity increases, resulting in a greater pressure drop. As a result, the most significant differences in the pressure field are primarily observed in the Adapter region.

**Figure 13:**
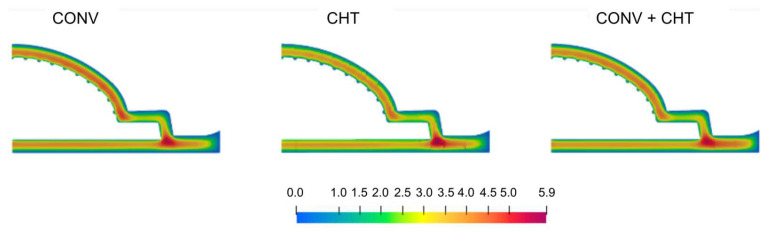
Comparison of outlet velocity field for the three case studies considered.

As evidenced by the velocity contours shown in Figure 13, at the outlet of the flow channel, the predicted flow fields were mostly similar among the three approaches, with some minor differences, especially between the CHT approach and the CONV and CONV+CHT approaches. When analyzing sections ES1, ES2, and ES3, it is evident that the CONV approach predicted higher velocities, while the CHT approach predicted the lowest velocities. This observation may initially seem counterintuitive, as higher temperatures in the CHT approach would typically promote higher velocities due to lower viscosities. However, upon closer examination of other sections, it becomes apparent that the slightly higher velocities predicted by the CONV approach in certain sections are offset by lower velocities in other sections. This effect is likely influenced by temperature’s impact on flow distribution. Since the flow rate remains constant in all cross sections, any increase in flow in certain sections must be compensated for by a decrease in flow in other sections. Therefore, while the CHT approach may result in lower velocities in some sections due to higher temperatures, it can also lead to slightly higher velocities in other sections, resulting in an overall balanced flow distribution.

**Figure 14:**
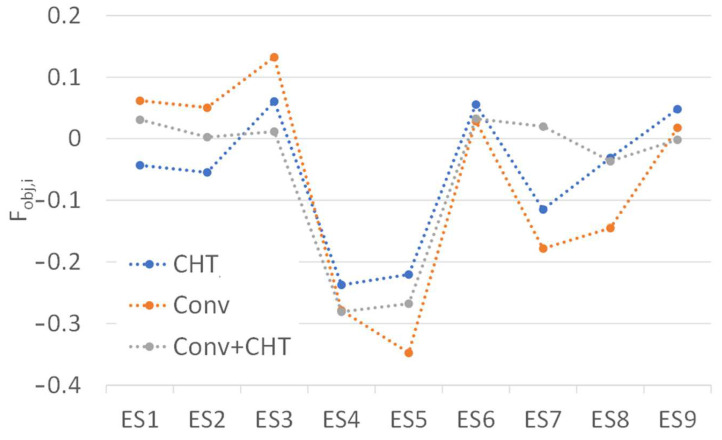
Comparison of F_Obj,i_, Equation (10), for all elemental sections and the three case studies.

When analyzing the results of the individual objective function (Equation (10)) for each elemental section, which is plotted in Figure 14, it is clear that in ES1, both the CONV and the CONV+CHT approaches predicted an excessive flow, while the CHT approach predicted an insufficient flow rate in that location. The CHT approach also predicted an insufficient flow rate at ES2, while the CONV+CHT approach predicted an almost perfect flow rate, and the CONV approach predicted an excessive flow. For ES3, it is noticeable that the CONV+CHT approach predicted an almost perfect flow rate, while the CONV approach predicted a significantly excessive flow. The CHT approach’s prediction was in between the predictions of the other two counterparts. All approaches predicted a lack of flow in ES4, with CONV+CHT and CONV being almost identical, while CHT was slightly higher and closer to the target flow due to the higher overall temperature. Similarly, in ES5, all approaches predicted a lack of flow, indicating the influence of temperature on flow; the higher temperature in the CHT approach resulted in a higher flow rate. Likewise, the higher wall temperature in the CONV+CHT approach led to higher flow rates compared to the CONV approach. For ES6, all approaches predicted a slightly higher flow rate, with CONV and CONV+CHT being almost equal and CHT slightly higher than the others. In the case of ES7, the mixed approach (CONV+CHT) predicted an excessive flow, while the other two approaches showed a deficit at the same location.

**Figure 15:**
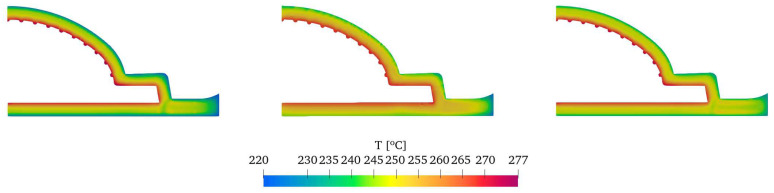
Comparison of outlet temperature field.

**Figure 16:**
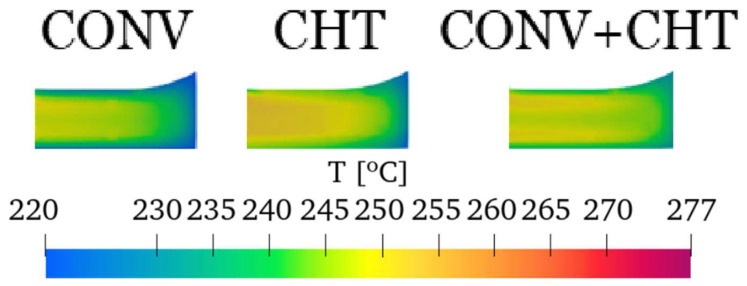
Corner temperature field detail.

An overall examination of the temperature distribution at the outlet, shown in Figure 15, reveals a consistent trend across all cases. However, upon closer inspection, noticeable differences in the temperature distribution among the cases can be observed. In sections ES1, ES2, ES3, ES4, ES5, and ES9, it is observed that the inner side of the temperature profile exhibits slightly higher temperatures in the CONV approach and lower temperatures in the CHT approach. This discrepancy arises from the lower temperatures of the outer walls applied in the CONV and CONV+CHT approaches, which promote higher viscosities and result in additional viscous dissipation near the inner wall. Figure 16 further highlights this effect, particularly in Section ES7, where imposing temperatures on the walls of the flow channel leads to lower temperatures within the flow channel itself. Finally, for ES8, the CONV approach predicted a lack of flow due to lower wall temperature, while the CONV+CHT and CHT approaches predicted excessive flow. At ES9, the flow rates are almost the same for all approaches.

In summary, across all elemental sections of the outlet, as presented in Figure 14, the results in all the different approaches follow the same trend, but some notable differences were identified. In general, higher temperature leads to a higher flow rate, influenced by the corresponding lower viscosity. However, due to continuity restrictions, the flow can only increase in one section if it decreases in another. Moreover, this intricate relationship may lead to counter-intuitive outcomes.

The above results highlight the importance of accurately calculating temperature distribution in extrusion processes, particularly in relation to their varying impact on different sections with varying levels of restriction. Larger, less restricted sections experience different temperature effects compared to smaller, highly restricted sections. Understanding these temperature dynamics is essential for optimizing profile extrusion processes. Accordingly, an accurate calculation of the temperature field is invaluable in comprehending many of the observed effects.

## 5. Conclusions

This study represents a significant advancement in the computational modeling of profile extrusion processes, addressing critical limitations in existing methodologies and providing valuable insights for process optimization and control. By introducing a novel simulation code integrated into the OpenFOAM computational library, capable of capturing incompressible, transient flow with a multi-region solver and realistic temperature control boundary conditions, we have achieved a more comprehensive understanding of temperature dynamics and flow distribution within extrusion dies.

Through rigorous industrial case studies and comparative analysis of different modeling approaches, including conventional, multi-region, and mixed strategies, we have demonstrated the effectiveness of the multi-region approach. By incorporating realistic temperature control boundary conditions based on thermocouple measurements and dynamic adjustment using PID algorithms, our simulations closely mimic real-world extrusion processes, offering invaluable insights for process optimization and control.

Our findings underscore the critical role of temperature in shaping flow distribution within extrusion dies, highlighting the importance of accurate temperature modeling for optimizing product quality and production efficiency. Furthermore, these studies showed that if the conventional approach usage of insulated boundary conditions for the mandrel surfaces provides a good approximation to the real case modeled by the CHT approach, this work provides practical guidance for industry practitioners and researchers, offering a robust framework for die design modeling and process improvement across various industrial applications.

Moving forward, future research efforts could focus on further validation and refinement of the proposed methodology, exploring additional case studies and incorporating more complex geometry and material properties. Additionally, ongoing advancements in computational techniques and high-performance computing offer exciting opportunities for enhancing the accuracy and efficiency of profile extrusion simulations.

## Figures and Tables

**Table 1:** Polycarbonate melt (fluid) properties.

Property	Description	Value	Units
$\eta_{0}$	Viscosity at null shear rate	5376	Pa·s
$\eta_{\infty }$	Viscosity at infinite shear rate	0	Pa·s
λ	Model parameter	0.0013	s
n	Power-law index	0.35	
$\frac{E}{R}$	Ratio between activation energy and the universal gas constant	13,951.59	K
$T_{0}$	Reference temperature	518.15	K
α	Thermal diffusivity	1.46 × 10^−7^	m^2^/s
*c_p_*	Specific heat capacity	1200	J/(kg·K)
*K*	Thermal conductivity	0.21	W/(m·K)

**Table 2:** Extrusion die (solid) properties.

Property	Description	Value	Units
α	Thermal diffusivity	3.33 × 10^−6^	m^2^/s
*K*	Thermal conductivity	16	W/(m·K)

**Table 3:** Conventional approach boundary conditions.

Patch	Pressure	Velocity	Temperature
Inlet	Null Normal Gradient	Fixed Value (0.282 m/min)	Fixed Value (245 °C)
Adapter	Null Normal Gradient	No Slip	Fixed Value (228 °C)
Die Land	Null Normal Gradient	No Slip	Fixed Value (220 °C)
Torpedo	Null Normal Gradient	No Slip	Zero Gradient
Symmetry	Symmetry	Symmetry	Symmetry
Outlet	Fixed Value (0 Pa)	Null Normal Gradient	Null Normal Gradient

**Table 4:** Multi-region approach boundary conditions.

Patch	Pressure	Velocity	Temperature
Inlet	Null Normal Gradient	Fixed Value (0.282 m/min)	Fixed Value (245°)
Adapter Heater	N/A	N/A	externalWallHeatFluxTemperaturePID Target T = 228 °C
Die Land Heater	N/A	N/A	externalWallHeatFluxTemperaturePID Target T = 220 °C
Adapter Thermocouple	N/A	N/A	Null Normal Gradient
Die Land Thermocouple	N/A	N/A	Null Normal Gradient
Die Wall	N/A	N/A	Natural Convection
Adapter Wall	N/A	N/A	Null Normal Gradient
Interface	Null Normal Gradient	No Slip	Mapped Wall
Symmetry	Symmetry	Symmetry	Symmetry
Outlet	Fixed Value (0 MPa)	Zero Gradient	Zero Gradient

**Table 5:** Mixed approach boundary conditions.

Patch	Pressure	Velocity	Temperature
Inlet	Null Normal Gradient	Fixed Value (0.282 m/min)	Fixed Value (245 °C)
Adapter	Null Normal Gradient	No Slip	Fixed Value (232 °C)
Die Land	Null Normal Gradient	No Slip	Fixed Value (230 °C)
Torpedo	Null Normal Gradient	No Slip	Null Normal Gradient
Symmetry	Symmetry	Symmetry	Symmetry
Outlet	Fixed Value (0 MPa)	Null Normal Gradient	Null Normal Gradient

## Data Availability

Data are contained within the article.
